# Metabolomics analysis of serum and urine in type 1 diabetes patients with different time in range derived from continuous glucose monitoring

**DOI:** 10.1186/s13098-024-01257-4

**Published:** 2024-01-19

**Authors:** Liyuan Ma, Jieying Liu, Mingqun Deng, Liyuan Zhou, Qian Zhang, Xinhua Xiao

**Affiliations:** 1 Peking Union Medical College Hospital, Chinese Academy of Medical Sciences & Peking Union Medical College, Beijing, 100730 China; 2 Department of Endocrinology, Key Laboratory of Endocrinology, Ministry of Health, Peking Union Medical College Hospital, Chinese Academy of Medical Sciences & Peking Union Medical College, Beijing, 100730 China; 3 Department of Medical Research Center, Peking Union Medical College Hospital, Chinese Academy of Medical Sciences & Peking Union Medical College, Beijing, 100730 China; 4 Department of Endocrinology, Beijing Hospital, National Center of Gerontology; Institute of Geriatric Medicine, Chinese Academy of Medical Sciences, Beijing, China; 5 Department of Endocrinology, Beijing Chao-Yang Hospital, Capital Medical University, Beijing, 100020 China

**Keywords:** Type 1 diabetes, Time in range, Glycemic variability, Metabolomics

## Abstract

**Background:**

Time in range (TIR), as an important glycemic variability (GV) index, is clearly associated with disease complications in type 1 diabetes (T1D). Metabolic dysregulation is also involved in the risks of T1D complications. However, the relationship between metabolites and TIR remains poorly understood. We used metabolomics to investigate metabolic profile changes in T1D patients with different TIR.

**Methods:**

This study included 85 T1D patients and 81 healthy controls. GV indices, including TIR, were collected from continuous glucose monitoring system. The patients were compared within two subgroups: TIR-L (TIR < 50%, n = 21) and TIR-H (TIR > 70%, n = 14). To screen for differentially abundant metabolites and metabolic pathways, serum and urine samples were obtained for untargeted metabolomics by ultra-performance liquid chromatography‒mass spectrometry. Correlation analysis was conducted with GV metrics and screened biomarkers.

**Results:**

Metabolites were significantly altered in T1D and subgroups. Compared with healthy controls, T1D patients had higher serum levels of 5-hydroxy-L-tryptophan, 5-methoxyindoleacetate, 4-(2-aminophenyl)-2,4-dioxobutanoate, and 4-pyridoxic acid and higher urine levels of thromboxane B3 but lower urine levels of hypoxanthine. Compared with TIR-H group, The TIR-L subgroup had lower serum levels of 5-hydroxy-L-tryptophan and mevalonolactone and lower urine levels of thromboxane B3 and phenylbutyrylglutamine. Dysregulation of pathways, such as tryptophan, vitamin B6 and purine metabolism, may be involved in the mechanism of diabetic complications related to glycemic homeostasis. Mevalonolactone, hypoxanthine and phenylbutyrylglutamine showed close correlation with TIR.

**Conclusions:**

We identified altered metabolic profiles in T1D individuals with different TIR. These findings provide new insights and merit further exploration of the underlying molecular pathways relating to diabetic complications.

**Supplementary Information:**

The online version contains supplementary material available at 10.1186/s13098-024-01257-4.

## Background

China has a high prevalence of diabetes, but only 49.4% of patients have well-controlled diabetes [1]. Type 1 diabetes (T1D), with an incidence of 3.6% in China, is caused by autoimmune-mediated destruction of islet β cells [2]. T1D patients have impaired glycemic regulation, facing a higher risk of acute and chronic complications from blood glucose fluctuations. Therefore, maintaining blood glucose homeostasis and preventing diabetic complications are crucial goals in diabetes management [3]. Glycemic variability (GV) is defined by fluctuations in glucose levels or related parameters over a specific time interval and is an essential metric for evaluating glycemic control in clinical practice [4]. HbA1c is the current gold standard for evaluating GV, but it can only reflect mean glycemic conditions over several months and is inaccurate in certain pathological states [4, 5]. Continuous glucose monitoring (CGM) is a novel approach that provides various metrics to quantify GV and can also assess the risk of hypo- and hyperglycemia, allowing for more precise monitoring of blood glucose [6].

Time in range (TIR), as a key CGM metric, indicates the percentage of time when blood glucose is within the target range (usually 70–180 mg/dL) [6]. The 2020 American Diabetes Association (ADA) “Standards of Medical Care in Diabetes” recommends the application of TIR for the assessment of glycemic control [5]. TIR has been used as an end point for many clinical trials [7]. There is a clear correlation between TIR and the onset and prognosis of diabetic complications. TIR is associated with an increased risk of diabetic retinopathy, peripheral neuropathy, and cardiovascular mortality in T2D patients [4, 8, 9]. For each 10% lower TIR in T1D patients, the hazard rates of retinopathy progression and microalbuminuria development were increased by 64% and 40%, respectively [7]. However, the exact mechanism behind the correlation between TIR and diabetic complications remains poorly understood, particularly in T1D patients. Therefore, further investigation is needed to gain a comprehensive understanding.

Metabolomics is a novel technology that can provide pathogenesis information of diabetes by analyzing metabolites and their interactions [10]. It studies small molecules, including organic acids, amino acids, carbohydrates and lipids, in cells, tissues or biofluids. Complex interactions between genes, proteins and environmental factors can be detected through downstream metabolites [10]. Amino acid alterations, such as branched-chain amino acids and aromatic amino acids, are related to the risk of diabetes [10], while lipidomic changes are associated with T1D autoimmunity [11]. Metabolites are also related to the onset and progression of diabetic complications. Lipidomic analysis revealed that sphingomyelin and phosphatidylcholine species were associated with diabetic nephropathy and all-cause mortality in T1D [12]. Impaired amino acid and TCA metabolism could be critical in cardiovascular autonomic neuropathy progression in T1D [13]. In addition, impaired TCA cycle metabolites in T1D lead to sensory nerve fiber loss in the skin and contribute to the progression of diabetic-induced peripheral neuropathy [14].

As we reviewed, no studies have explored the association between TIR and metabolomics. The present study combined CGM and metabolomics technology to analyze the metabolite characteristics of T1D patients, including subgroups divided by TIR and healthy individuals. Then, we analyzed the relationship between GV indices and identified differentially abundant metabolites.

## Methods

This study recruited a total of 85 T1D patients and 81 healthy controls. Patients (ranging in age from 18 to 65) diagnosed with T1D according to the 1999 World Health Organization criteria were included from the Endocrinology Department of Peking Union Medical College Hospital (PUMCH) between October 2018 and March 2019. All patients had a stable dose of insulin usage for more than 3 months (dose change < 10%). The exclusion criteria are in the Additional file 1. Observations of the Flash Glucose Monitor (FGM) were performed for 14 days to collect TIR and other GV metrics calculated by Excel and Easy GV version 9.0 R2 (Oxford University). All patients maintained a normal drug treatment schedule throughout the study. According to the recommended target goals for TIR of > 70% and > 50% for general and high-risk individuals with diabetes, respectively, T1D patients were divided into 3 subgroups with different TIR: low TIR (TIR-L, TIR < 50%, n = 21), high TIR (TIR-H, TIR > 70%, n = 14) and moderate TIR (TIR-M, 50% ≤ TIR ≤ 70%, n = 50) groups. In order to screen metabolites having more significant difference and closer relation with glycemic variability, TIR-L and TIR-H subgroups were used for further metabolomics comparison. The study was approved by the Ethics Committee of PUMCH (No. 13275). All subjects signed the informed consent form.

### Clinical data collection

Questionnaires were used to acquire gender, age, the onset of diabetes, duration of diabetes, complications of diabetes, insulin regimen, drug use, family history, smoking and drinking history, exercise frequency, and other information. Physical examinations were performed to obtain general conditions, blood pressure, and BMI. Laboratory tests of HbA1c, fasting and postprandial blood glucose (FBG, PBG), C-peptide, and other biochemical indices, such as cholesterol and triglycerides, were tested by the laboratory department in PUMCH.

### Untargeted metabolomics assay

Serum and urine samples were collected at the beginning of 2 weeks of FGM observation and stored at − 80 °C. An untargeted metabolomics assay was performed by ultra-performance liquid chromatography tandem mass spectrometry (UPLC‒MS). A Waters H-class UPLC system was used. The detailed conditions are shown in the Additional file 1.

### Statistical analysis, biomarker identification, and metabolic pathway analysis

The raw data obtained by the UPLC-LTQ Orbitrap were imported to Progenesis QI software (Version 2.0, Nonlinear Dynamics, UK) for processing. The processing procedure includes peak alignment, identification and correction. The output is a three-dimensional matrix consisting of retention times and exact mass-to-charge ratios of peaks, sample names and peak intensities or areas. The matrix was analyzed following SIMCA-P software 14.0 (Umetrics AB, Umea, Sweden) to identify the group difference via principal component analysis and screen the potential biomarkers via orthogonal partial least-squares discrimination analysis (OPLS-DA). Variable importance in the project (VIP) values, t test, fold change (FC) and hierarchical cluster analysis were analyzed by MetaboAnalyst 5.0. VIP > 1 and P value < 0.05 were used as conditions for filtering potential biomarkers. Afterward, biochemical databases, including HMDB (Human Metabolome Database) and KEGG (Kyoto Encyclopedia of Genes and Genomes), were used to identify and confirm potential biomarkers. Pathway analysis was performed using MetaboAnalyst 5.0 by uploading the identified compound names to the program to calculate the impact value and p value of enriched pathways.

Other data analysis was performed using SPSS 22.0 software (IBM Corp., Armonk, N.Y., USA). Continuous data are expressed as the mean ± standard deviation (SD) or median (interquartile range). Student’s *t* test or Mann-Whitey *U* test was used to compare two groups, while ANOVA was used for three groups. Categorical variables were expressed as numbers (percentages) and compared using the chi-square test. Correlation analysis was performed using binary logistic regression, Spearman, partial correlation, and multiple linear regression analyses. Statistical significance was determined by a p value of < 0.05. Graph Pad Prism 8.4.3 (GraphPad Software, San Diego, California, USA) was used to perform receiver operating characteristic (ROC) curve analysis.

## Results

### Clinical characteristics

A total of 85 patients with T1D and 81 healthy subjects were enrolled in this study. The demographic, anthropometric, and biochemical characteristics of all participants are presented in Additional file 1: Table S1. There was no significant difference in sex, age or BMI between the two groups. The T1D patients showed significantly higher HbA1c levels than the healthy controls, which is typical of diabetic patients. Control subjects had significantly higher TG and LDL-C and lower HDL-C levels than T1D patients.

The characteristics of T1D patients with different TIR are presented in Tables 1and2. Among 85 T1D patients, the median TIR was 59.50 (18.45) %. Only 16.47% of patients had TIR > 70%. HbA1c, FBG and PBG were significantly lower in T1D patients who had higher TIR. Comparing the TIR-L group with the TIR-H group, there was no significant difference in sex, age, BMI, blood pressure, serum lipid, ALT, eGFR, complications, family history, smoking and alcoholic history, exercise frequency or oral medications. The fasting serum C-peptide concentration of TIR-H patients was significantly higher than that of TIR-L patients. TIR-L patients had an earlier age of onset, longer duration of T1D, and larger daily insulin dose than TIR-H patients. For glucose indices, TIR-L patients had significantly higher HbA1c [9.00 (1.70)% vs. 6.80 (1.40)%, P < 0.001], FBG and PBG than TIR-H patients. Comparing CGM metrics (Table 2), TIR-L patients had lower TBR1 and higher TAR (including TAR1 and TAR2), CV, SD, MG, MODD, MAGE, MAG, CONGA, HBGI, GRADE, JINDEX, LI and MVALUE than TIR-H subjects.

**Table 1:** Clinical characteristics of the three TIR subgroups in T1D patients

Variable	Overall (N = 85)	TIR-L (N = 21)	TIR-M (N = 50)	TIR-H (N = 14)	P-value (3 subgroup)	P-value (TIR-L vs. TIR-H)
Gender (F/M)	55/30	13/8	33/17	9/5	0.950	1.000
Age (y.o)	35.00(18.00)	30.00(25.50)	36.00(14.00)	35.50(32.75)	0.323	0.152
Age at onset (y.o)	27.96(19.64)	25.71(26.71)	26.48(19.81)	33.24(27.77)	0.056	**0.043**
Duration of T1D (years)	6.60(10.61)	8.85(9.45)	8.22(12.33)	3.40(5.23)	**0.009**	**0.024**
BMI (kg/m^2^)	21.16(3.65)	21.20(5.17)	21.10(4.34)	21.37(3.50)	0.584	0.602
SBP (mmHg)	114.00(21.00)	113.00(20.00)	111.50(21.25)	118.50(28.25)	0.897	0.625
DBP (mmHg)	70.00(15.50)	70.00(12.50)	70.00(18.00)	68.00(12.25)	0.713	0.354
TC (mmol/L)	4.63(1.14)	4.47(1.47)	4.67(1.26)	4.69(0.77)	0.874	0.893
TG (mmol/L)	0.54(0.30)	0.56(0.22)	0.55(0.34)	0.48(0.28)	0.474	0.138
HDL-C (mmol/L)	1.57(0.69)	1.46(0.72)	1.57(0.69)	1.70(0.69)	0.714	0.490
LDL-C (mmol/L)	2.43(0.99)	2.46(0.94)	2.42(1.03)	2.35(0.74)	0.703	0.400
ALT (U/L)	15.00(8.00)	16.00(7.00)	15.00(8.25)	18.00(16.00)	0.497	0.266
Cr (umol/L)	59.70(16.00)	59.00(13.50)	61.50(17.75)	57.00(13.85)	0.459	0.555
eGFR (ml/min/1.73m^2^)	167.19(61.69)	176.12(84.90)	159.23(65.21)	168.92(49.61)	0.500	0.459
HbA1c (%)	7.40(1.75)	9.00(1.70)	7.15(1.20)	6.80(1.40)	** < 0.001**	** < 0.001**
FBG (mmol/L)	8.80(6.50)	12.50(6.20)	8.10(6.98)	7.35(3.38)	**0.002**	**0.001**
1 h-BG (mmol/L)	16.60(8.25)	19.70(5.80)	16.50(10.00)	13.80(4.93)	**0.001**	** < 0.001**
2 h-BG (mmol/L)	19.50(9.95)	23.40(8.60)	19.15(10.63)	16.25(10.35)	**0.011**	**0.006**
FCP (ng/mL)	0.00(0.29)	0.00(0.20)	0.00(0.15)	0.34(0.46)	**0.015**	**0.025**
Family history, N(%)	28(32.9)	7(33.3)	17(34.0)	4(28.6)	1.000	1.000
Diabetic complications, N(%)	13(15.3)	5(23.8)	7(14.0)	1(7.1)	0.401	0.366
Smoke, N(%)	10(11.8)	5(23.8)	4(8.0)	1(7.1)	0.189	0.366
Drinking alcohol, N(%)	17(20.0)	5(23.8)	10(20.0)	2(14.3)	0.866	0.676
Regular exercise, N(%)	17/8/60	5/3/13	9/2/39	3/3/8	0.185	0.894
Insulin pump, N(%)	21(24.7)	4(19.0)	14(28.0)	3(21.4)	0.779	1.000
Daily Insulin dose (U/d)	34.00(16.50)	36.00(15.03)	34.08(19.45)	26.50(13.00)	**0.004**	**0.003**
Average daily Insulin dose (U/kg/d)	0.59(0.23)	0.63(0.21)	0.58(0.24)	0.50(0.24)	**0.016**	**0.016**
Oral medications						
AGI, N(%)	16(18.8)	4(19.0)	8(16.0)	4(28.6)	0.587	0.685
Metformin, N(%)	20(23.5)	7(33.3)	10(20.0)	3(21.4)	0.459	0.704

Bold text represents P value < 0.05

n (%); Median (IQR); Fisher exact test; Kruskal–Wallis H test; Mann-Whitey *U* test

TIR, time in range; TIR-H, high TIR group; TIR-M, moderate TIR group; TIR-L, low TIR group; BMI, body mass index; SBP, systolic blood pressure; DBP, diastolic blood pressure; TC, total cholesterol; TG, triglyceride; HDL-C, high density lipoprotein cholesterol; LDL-C, low density lipoprotein cholesterol; ALT, alanine transaminase; Cr, creatine; eGFR, estimated glomerular filtration rate; HbA1c, glycosylated hemoglobin; FBG, fasting blood glucose; FCP, fasting C- peptide; AGI, α-glycosidase inhibitor

**Table 2:** CGM metrics of the three TIR subgroups in T1D patients

Variable	Overall (N = 85)	TIR-L (N = 21)	TIR-M (N = 50)	TIR-H (N = 14)	P-value (3 subgroup)	P-value (TIR-L vs. TIR-H)
TIR (%)	59.50(18.45)	36.50(20.65)	61.95(8.10)	76.50(14.33)	** < 0.001**	** < 0.001**
TBR (%)	7.50(15.50)	3.10(3.90)	11.30(17.45)	5.60(7.05)	**0.001**	0.178
TBR1 (%)	4.80(6.15)	2.20(2.65)	7.75(7.85)	4.30(4.23)	** < 0.001**	**0.019**
TBR2 (%)	1.70(7.55)	1.00(1.45)	3.75(9.38)	0.90(2.90)	**0.010**	0.748
TAR (%)	28.20(30.40)	60.60(21.21)	24.10(18.38)	14.30(14.90)	** < 0.001**	** < 0.001**
TAR1 (%)	19.60(16.50)	33.60(12.95)	18.75(14.80)	11.75(11.63)	** < 0.001**	** < 0.001**
TAR2 (%)	5.60(12.20)	29.90(24.20)	4.35(6.45)	0.30(3.63)	** < 0.001**	** < 0.001**
CV (%)	40.10(10.55)	37.10(13.80)	42.45(10.58)	33.60(6.08)	** < 0.001**	**0.028**
SD (mmol/L)	3.29(0.98)	4.40(1.30)	3.26(0.73)	2.61(0.87)	** < 0.001**	** < 0.001**
MG (mmol/L)	8.08(2.72)	11.48(2.63)	7.75(2.10)	7.19(1.43)	** < 0.001**	** < 0.001**
MODD (mmol/L)	3.25(1.22)	4.45(2.00)	3.35(1.01)	2.07(1.09)	** < 0.001**	** < 0.001**
MAGE (mmol/L)	7.89(2.27)	9.72(3.67)	7.58(1.92)	6.22(2.06)	** < 0.001**	** < 0.001**
MAG (mmol/L)	1.80(0.41)	2.03(0.63)	1.78(0.45)	1.73(0.34)	** < 0.001**	** < 0.001**
CONGA	7.53(2.64)	10.58(2.58)	7.12(1.79)	6.49(1.43)	** < 0.001**	** < 0.001**
LBGI	5.49(5.79)	5.19(4.92)	6.84(5.72)	3.71(2.99)	**0.013**	0.064
HBGI	8.60(6.19)	20.08(9.05)	8.13(3.57)	5.14(3.48)	** < 0.001**	** < 0.001**
GRADE	6.02(5.25)	13.47(6.35)	5.89(2.48)	3.69(3.26)	** < 0.001**	** < 0.001**
JINDEX	41.72(27.70)	89.30(33.98)	39.57(17.58)	30.42(13.99)	** < 0.001**	** < 0.001**
LI (mmol/L^2^/h·week^−1^)	3.93(2.09)	5.43(2.96)	3.92(1.73)	2.99(1.40)	** < 0.001**	** < 0.001**
MVALUE (mmol/L)	14.54(11.67)	32.37(16.14)	14.28(6.26)	6.62(4.67)	** < 0.001**	** < 0.001**

Bold text represents P value < 0.05

Median (IQR); Kruskal–Wallis H test; Mann-Whitey *U* test

CGM, Continuous glucose monitoring; TIR, time in range; TIR-H, high TIR group; TIR-M, moderate TIR group; TIR-L, low TIR group; TBR, time below range; TAR, time above range; CV, coefficient of variation; SD, standard deviation; MG, mean glucose; MODD, mean of daily differences; MAGE, mean amplitude of glycemic excursions; MAG, mean absolute glucose; CONGA, continuous overall net glycemic action; LBGI, low blood glucose index; HBGI, high blood glucose index; GRADE, glycemic risk assessment diabetes equation; LI, lability index

### Metabolite metabolomic differences between T1D patients and controls

**Fig. 1:**
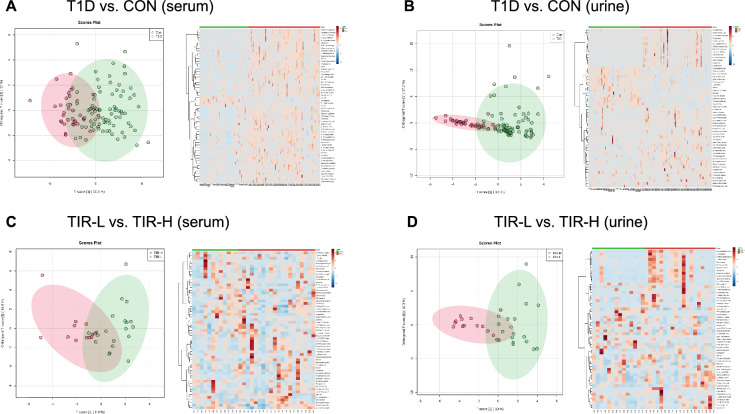
OPLS-DA plots and hierarchical clustering analysis of differentially abundant metabolites. **A** T1D patients compared with control serum samples. **B** T1D patients compared with control urine samples. **C** TIR-H versus TIR-L subgroup of serum samples. **D** TIR-H versus TIR-L subgroup of urine samples. The left figures are OPLS-DA score plots. The horizontal axis represents intergroup differences, while the vertical axis represents intragroup differences. The right figures are heatmaps of hierarchical clustering analysis. The shift from blue to red indicates an increase in content. Each row represents a potential biomarker, and each column represents a sample. TIR, time in range; TIR-H, high TIR group; TIR-L, low TIR group; OPLS-DA, orthogonal partial least-squares discrimination analysis

**Fig. 2:**
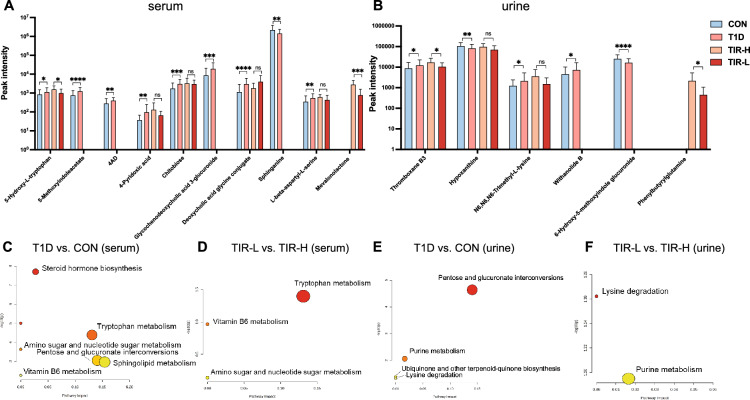
The peak intensity of the 16 potential biomarkers in T1D and subgroups and the pathway analysis of differentially abundant metabolites. The comparison of peak intensity of potential biomarkers in serum (**A**) and urine (**B**) was performed using a *t* test. The asterisk represents the P value, and one asterisk, two asterisks and three asterisks represent P < 0.05, P < 0.01 and P < 0.001, respectively. **C–F** Pathway analysis of serum and urine samples between T1D versus control and TIR-L versus TIR-H subgroups. Each bubble in the plot depicts a metabolic pathway, with size reflecting enrichment level. TIR, time in range; TIR-H, high TIR group; TIR-L, low TIR group; 4AD, 4-(2-aminophenyl)-2,4-dioxobutanoate

The OPLS-DA plots in Fig. 1A and B show that the metabolomics was different between T1D patients and controls in serum and urine samples. A total of 54/440 serum and 45/158 urine differentially abundant metabolites were identified between them. The heatmaps of the hierarchical clustering analysis of differentially abundant metabolites are shown in Fig. 1A and B. Additional file 1: Tables S2 and S3 summarize the 15 serum and 7 urine differentially abundant metabolites included in both HMDB and KEGG in order of P value. Figure 2C and E and Additional file 1: Table S6 demonstrate the pathway analysis. There were 7 and 4 different metabolic pathways in serum and urine, mainly including tryptophan metabolism, vitamin B6 metabolism, sphingolipid metabolism, amino sugar and nucleotide sugar metabolism, pentose and glucuronate interconversions, lysine degradation, and purine metabolism. Fourteen potential biomarkers in total were selected with a P value < 0.05. Figure 2A and B demonstrate the comparison of peak intensity, and Table 3 shows the P value, FC, VIP value, and pathway involved in each metabolite. Performing a binary logistic regression analysis of 14 potential biomarkers with age, sex, BMI, TG, HDL-C, and LDL-C as confounding factors, it was found that elevated 5-hydroxy-L-tryptophan, 5-methoxyindoleacetate, 4-(2-aminophenyl)-2,4-dioxobutanoate (4AD), 4-pyridoxic acid, deoxycholic acid glycine conjugate, and decreased sphinganine in serum, as well as elevated thromboxane B3 in urine, can be used as T1D predictive factors.

**Table 3:** The logistic regression and ROC curve analysis of the 16 potential biomarkers

Metabolite	Pathway	log2 (FC)	P value	VIP		ORa		95%CI		AUC
T1D vs. CON (serum)										
5-Hydroxy-L-tryptophan	Tryptophan metabolism	0.4154	**0.026**	0.8087		**1.777**		1.059–2.981		0.6236
5-Methoxyindoleacetate	Tryptophan metabolism	0.7270	** < 0.001**	**1.0660**		**6.279**		2.754–14.315		0.7358
4-(2-Aminophenyl)-2,4-dioxobutanoate	Tryptophan metabolism	0.4918	**0.001**	0.9853		**3.515**		1.689–7.312		0.7196
4-Pyridoxic acid	Vitamin B6 metabolism	1.3753	**0.006**	0.7594		**3.939**		1.596–9.721		0.7263
Chitobiose	Amino sugar and nucleotide sugar metabolism	0.8174	** < 0.001**	0.9247		**2.755**		1.535–4.943		0.7336
Glycochenodeoxycholic acid 3-glucuronide	Pentose and glucuronate interconversions	1.1391	**0.001**	0.7707		**2.342**		1.528–3.590		0.7565
Deoxycholic acid glycine conjugate	Bile acid biosynthesis	1.4009	** < 0.001**	0.9400		**2.080**		1.318–3.824		0.7536
Sphinganine	Sphingolipid metabolism	− 0.6070	**0.001**	0.8390		**0.348**		0.163–0.742		0.6588
L-beta-aspartyl-L-serine	–	0.6399	**0.003**	0.7076		**1.786**		1.065–2.993		0.7064
T1D vs. CON (urine)										
Thromboxane B3	–	0.4821	**0.036**	0.8661		**1.827**		1.022- 3.266		0.6669
Hypoxanthine	Purine metabolism	− 0.3541	**0.009**	**1.1453**		–		–		0.6415
N6,N6,N6-Trimethyl-L-lysine	Lysine degradation	0.7730	**0.048**	0.4991		–		–		0.5994
Withanolide B	Ubiquinone and other terpenoid-quinone biosynthesis	0.6992	**0.041**	0.6403		**1.802**		1.233- 2.633		0.6464
6-Hydroxy-5-methoxyindole glucuronide	Pentose and glucuronate interconversions	− 0.6375	** < 0.001**	**1.5086**		**0.271**		0.135–0.545		0.6961
TIR-L vs. TIR-H (serum)										
5-Hydroxy-L-tryptophan	Tryptophan metabolism	− 0.6442	**0.040**	**1.6627**		–		–		0.7250
4-Pyridoxic acid	Vitamin B6 metabolism	− 0.9901	0.109	**1.8041**		–		–		0.6542
Deoxycholic acid glycine conjugate	Bile acid biosynthesis	1.1396	0.113	**1.2084**		–		–		0.6375
Chitobiose	Amino sugar and nucleotide sugar metabolism	− 0.1320	0.736	**1.2366**		–		–		0.5125
Mevalonolactone	–	− 1.8308	** < 0.001**	**2.6453**		**0.054**		0.008–0.368		0.9375
L-beta-aspartyl-L-serine	–	− 0.4808	0.113	**1.3370**		–		–		0.7292
TIR-L vs. TIR-H (urine)										
Thromboxane B3	–	− 0.7022	**0.024**	**1.5800**		0.201		0.039–1.023		0.7218
N6,N6,N6-Trimethyl-L-lysine	Lysine degradation	− 1.2279	0.055	**1.1390**		**0.304**		0.101–0.908		0.7068
Hypoxanthine	Purine metabolism	− 0.4618	0.064	**1.0109**		**0.075**		0.008–0.739		0.7030
Phenylbutyrylglutamine	–	− 2.2585	**0.027**	**1.3021**		**0.144**		0.033–0.633		0.7782

TIR, time in range; TIR-H, high TIR group; TIR-L, low TIR group; FC, fold change; VIP, variable importance in the project; OR, odds ratio; CI, confidence interval; ROC, receiver operating characteristic; AUC, area under curve

^a^Bold text of OR represents significance(P < 0.05)

### Metabolomic differences between T1D subgroups with different TIR

OPLS-DA plots of T1D subgroups of TIR-H versus TIR-L in serum and urine samples are presented in Fig. 1C and D, which show that the metabolomics profiles were different between them. The TIR-L versus TIR-H group had 19/252 and 30/121 differentially abundant metabolites identified in the serum and urine samples, respectively. Figure 1C and D also show the heatmaps of the hierarchical clustering analysis of differentially abundant metabolites. Additional file 1: Tables S4 and S5 list 15 serum and 19 urine differentially abundant metabolites either included in HMDB or KEGG identified by the TIR group in order of VIP value. Figure 2D and F and Additional file 1: Table S6 demonstrate the pathway analysis. Comparing TIR-L with the TIR-H subgroup, tryptophan metabolism, vitamin B6 metabolism, amino sugar and nucleotide sugar metabolism in serum (Fig. 2D), and lysine degradation and purine metabolism in urine (Fig. 2F) were 5 different metabolic pathways. Ten potential biomarkers in total were selected with a P value < 0.05 or VIP > 1, as demonstrated in Fig. 2A and B and Table 3. Table 3 also demonstrates the binary logistic regression analysis of 10 potential biomarkers in the TIR comparison group, with age, sex, BMI, duration of disease and insulin dosage as confounding factors. It was found that decreased mevalonolactone in serum and phenylbutyrylglutamine, hypoxanthine, N6, N6, and N6-trimethyl-L-lysine in urine can be used as low-level TIR predictive factors.

### Correlation analysis of potential biomarkers and GV metrics

**Fig. 3:**
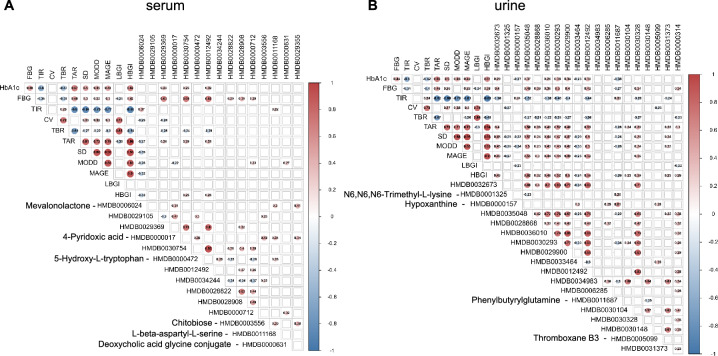
Spearman correlation analysis between differentially abundant metabolites and glycemic variability index. **A** Analysis of serum samples. **B** Analysis of urine samples. The correlation with a P value < 0.05 is shown with a circle. The number in the circle represents the correlation value. Red and blue represent positive and negative correlations, respectively. The darker the color and the larger the circle, the greater the absolute value of the correlation coefficient. TIR, time in range; TBR, time below range; TAR, time above range; CV, coefficient of variation; SD, standard deviation; MODD, mean of daily differences; MAGE, mean amplitude of glycemic excursions; LBGI, low blood glucose index; HBGI, high blood glucose index

Spearman correlation analysis of GV metrics (TIR, TBR, TAR, CV, SD, MODD, MAGE, LBGI, HBGI) with 15 serum and 19 urine differential metabolites screened by the TIR group was showed in Fig. 3. To further investigate the correlation of metabolites with TIR, partial correlation analysis and multilinear regression analysis adjusting for sex, age, BMI, duration of disease, and insulin dosage as confounding factors were conducted (Additional file 1: Table S7). In serum, mevalonolactone was positively related to TIR (R = 0.367, P = 0.001) and negatively related to CV, TAR, SD, MODD, MAGE, and HBGI (R = − 0.252, − 0.269, − 0.350, − 0.285, − 0.316, − 0.321, P = 0.023, 0.015, 0.001, 0.010, 0.004, 0.003). Mevalonolactone was still significantly correlated with TIR (R = 0.320, P = 0.005) by partial correlation analysis and is an independent predictive factor for TIR in multilinear regression analysis (β = 0.348, P = 0.001, F = 10.902, R = 0.348). In urine, TIR was positively correlated with hypoxanthine and phenylbutyrylglutamine (R = 0.244, 0.329, P = 0.032, 0.003) but negatively correlated with the other 8 metabolites. Partial correlation analysis of these 10 metabolites demonstrated that hypoxanthine and phenylbutyrylglutamine were still significantly correlated with TIR (R = 0.232, 0.308, P = 0.048, 0.008). Multilinear regression analysis showed that phenylbutyrylglutamine is an independent predictive factor for TIR (β = 0.348, P = 0.002, F = 10.462, R = 0.348). Besides, L-beta-aspartyl-L-serine was also positively related to TIR (R = 0.222, P = 0.046). Deoxycholic acid glycine conjugate was positively correlated with MODD (R = 0.270, P = 0.015).

### Biomarker analysis

**Fig. 4:**
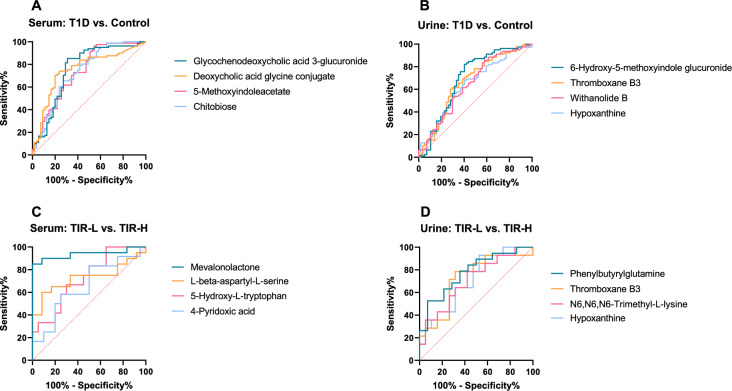
The ROC curve of the potential biomarkers. Analysis of potential biomarkers to discriminate T1D from controls in serum (**A**) and urine (**B**) samples and to discriminate TIR-H from the TIR-L subgroup in serum (**C**) and urine (**D**) samples. ROC, receiver operating characteristic; TIR, time in range; TIR-H, high TIR group; TIR-L, low TIR group

The 16 potential biomarkers were analyzed by ROC curve analysis. Table 3 summarizes the area under the curve (AUC) values. The metabolites with the top four AUC rankings in the list shown in Fig. 4 were selected for combined analysis. The panels obtained AUCs of 0.779 (95% CI 0.677–0.889) and 0.715 (95% CI 0.603–0.830) in serum and urine, respectively, to distinguish T1D from healthy subjects. For TIR-L versus TIR-H, the panel in serum reached an AUC of 0.793 (95% CI 0.440–0.990), lower than that of mevalonolactone as a biomarker alone (AUC = 0.938), and four urine metabolites obtained an AUC of just 0.664 (95% CI 0.400–0.860), without better performance than phenylbutyrylglutamine alone (AUC = 0.778).

## Discussion

T1D is a chronic autoimmune disease with insulin deficiency due to β-cell destruction. Metabolomics systematically identifies and quantitates metabolites from biological systems. Certain metabolites and metabolic pathways are associated with risks of T1D onset and complications [10]. T1D complications are also strongly correlated with glycemic fluctuation. Investigation of metabolite profile changes affected by glycemic fluctuation can assist in understanding the mechanism of T1D complications.

First, this study supported that TIR is a reliable GV indicator. We demonstrated that T1D patients with lower levels of TIR have an earlier age of onset, a longer duration of disease, a larger daily insulin dose, and higher levels of blood glucose, HbA1c, fasting serum C-peptide, and CGM indices, representing greater glycemic fluctuation. These results strengthen the use of TIR as an indicator to evaluate glycemic control and to assist in the management of T1D.

In addition to comparison with healthy individuals, we showed how the metabolic profiles of T1D patients with different TIR varied for metabolomics analysis. Tryptophan metabolism, vitamin B6 metabolism and purine metabolism were the major changed pathways, which may have an impact on GV and be involved in the development of diabetic complications.

This study showed that three metabolites in the tryptophan metabolism pathway were increased in T1D, including 5-hydroxy-L-tryptophan, 5-methoxyindoleacetate, and 4AD. Among them, 5-hydroxy-L-tryptophan was reduced in the TIR-L subgroup. Tryptophan (TRP) is essential for humans and is mainly involved in two metabolic pathways: serotonin synthesis and the kynurenine pathway (KP). The majority of TRP is degraded through KP, which produces kynurenine (KYN) and kynurenic acid (KYA) and finally synthesizes NAD^+^ for energy production [15]. T1D development can be influenced by modifications in TRP metabolism. Reduced plasma free L-tryptophan levels may impact neural signaling and serotonin metabolism related to neuropsychiatric disorders [16]. Mouse models show TRP metabolism changes at the early stage of T1D disease progression [15]. Another study found increased plasma TRP and a decreased KYN/TRP ratio in T1D patients. They proposed that TRP metabolism may contribute to T1D autoimmunity through the augmentation of autoimmune-induced pancreatic cell apoptosis and the reduction in TRP consumption influenced by the gut microbiome [17]. An in vitro study suggested that dendritic cells regulate TRP metabolism to inhibit T lymphocyte function, potentially affecting T1D autoimmunity development [18]. 5-Hydroxy-L-tryptophan, a precursor of serotonin, also increases insulin release in pancreatic β cells [19]. Supplementation with 5-hydroxytryptophan rescued glucose-induced insulin secretion in defect pancreatic β-cells [20]. Additionally, 5-hydroxy-L-tryptophan is decreased in early pregnancy serum among gestational diabetes mellitus [21], and it induces more rapid hypoglycemia than dosing tryptophan in rats [22]. This study showed higher levels of 5-hydroxy-L-tryptophan in the T1D and TIR-H subgroups. T1D individuals may have compensatory mechanisms that increase 5-hydroxy-L-tryptophan to release more insulin in pancreatic β cells. The TIR-H subgroup may have better compensatory mechanisms, resulting in higher levels of 5-hydroxy-L-tryptophan. 5-Methoxyindoleacetate is a downstream metabolite of serotonin, and 4AD is an intermediate in the conversion of KYN to KYA [23]. Thus, metabolic changes in these two metabolites may be associated with the abovementioned key reactions. Diabetic rats fed with advanced glycation end-products had significantly higher levels of oxidative stress and lower 5-methoxyindoleacetate levels in serum and urine [24]. In rats, 5-methoxyindoleacetate has been associated with ischemic stroke [25]. Interestingly, 5-methoxyindoleacetate and 4AD were higher in T1D patients in this study. The underlying mechanisms are not clear and require further investigation.

The T1D and TIR-L subgroups had altered vitamin B6 metabolism in this study. Vitamin B6 metabolism is impaired at an early stage in T1D [26], and vitamin B6 deficiency is an independent risk factor for diabetic complications, including cardiovascular, cerebrovascular, and peripheral vascular disease [27]. Supplementation with vitamin B6 normalized endothelial dysfunction in T1D [27]. 4-Pyridoxic acid (4-PA) is the vitamin B6 degradation product. A study showed that plasma and urinary excretion of 4-PA were increased in diabetic patients (P < 0.001) and positively correlated with HbA1c and diabetes duration, indicating that the degradation of vitamin B6 may be related to metabolic dysfunction in hyperglycemia and increases in severity as diabetes progresses [28]. In this study, serum 4-PA was higher in T1D, which is in line with previous findings. However, it tended to be lower in the TIR-L group. The activity of enzymes degrading vitamins may explain this result, which was increased in T1D patients but with better function in the TIR-H subgroup, resulting in higher degradation products. Further studies are needed to confirm this hypothesis.

Hypoxanthine in urine was markedly reduced in T1D and tended to be lower in the TIR-L subgroup with VIP > 1. It was also an independent predictive factor and positively related to TIR. Hypoxanthine is a crucial substance in purine metabolism. The conversion of hypoxanthine to xanthine and then to uric acid (UA) requires xanthine oxidase (XO) catalysis and generates reactive oxygen species (ROS). Hypoxanthine and its oxidation product can be used as potential markers for monitoring the oxidative state [29]. Studies have explored the association between purine metabolism and diabetes. Nonobese diabetic mice had higher xanthine levels in the pancreas [15]. Purine metabolism disorder is correlated with an elevated risk of diabetic nephropathy (DN), supported by evidence of elevated UA in both rats and T1D patients [30, 31]. XO is an important source of hyperglycemia-induced ROS production in skeletal muscle [32]. XO can directly harm kidney cells by oxidative damage and indirectly induce inflammation by activating the NF-κB signaling pathway in DN rats [31]. The drop in hypoxanthine observed in T1D and the positive correlation with TIR may indicate that more hypoxanthine is metabolized to downstream metabolites, causing more ROS in T1D with unstable glucose control.

In addition to purine metabolism, other differentially abundant metabolites (thromboxane B3 [33], chitobiose [34], withanolide B [35]) and metabolic pathways (amino sugar and nucleotide sugar metabolism, pentose and glucuronate interconversions [36]) we screened are also possibly related to oxidative stress and inflammation, which is consistent with the proposed mechanism between GV and the risk of complications from T1D [37]. A long duration of blood glucose fluctuations leads to the accumulation of ROS, affecting the expression of related genes and resulting in pathophysiological changes, thereby increasing the risk of complications [38].

In addition to the metabolites implicated in these pathways, we discovered some rarely reported metabolites that performed well as potential biomarkers, such as mevalonolactone and phenylbutyrylglutamine, which are positively related to TIR and can be independent predictive factors. Through the mevalonate pathway, mevalonolactone is a precursor for the biosynthesis of various steroids and isoprenoids. Inhibitors of this pathway, such as statins, which inhibit HMG-CoA reductase, can reduce blood cholesterol levels. The mevalonate pathway also regulates the development and survival of brown adipocytes [39]. Mevalonolactone may therefore be an important factor involved in GV by affecting cholesterol synthesis. Phenylbutyrylglutamine is a metabolite of phenylbutyrate that is used to treat thalassemia, cancer, etc. [40] but has not yet been related in any reports to diabetes.

We observed that T1D patients had elevated deoxycholic acid glycine conjugate, which is a secondary bile acid (BA) produced in the liver by the conjugation of deoxycholate with glycine. BAs can help with the excretion, absorption, and transport of lipids in the liver and intestines. Dysregulated BA metabolism contributes to the risk and pathogenesis of T1D [41]. In addition, T1D patients in this study showed low TG and LDL-C levels and high HDL-C levels, possibly due to differences in insulin distribution compared to healthy individuals. Most of the exogenously supplied insulin in T1D is distributed in circulation, and little reaches the liver, leading to reduced TG and LDL synthesis. Alternatively, higher levels of BAs in T1D patients may lower serum lipids. However, further investigation is needed to understand lipid metabolism in T1D.

Our study has several strengths and limitations. This research combined two novel technologies, metabolomics and CGM, which are rarely reported. The exploration of the changes in metabolism affected by glycemic fluctuations in T1D patients provides insights for further research into the mechanism of complications. However, the UPLC‒MS method is still challenging in identifying exact chemical structures from peak data. The targeted metabolomics method can be used for further verification. We did not include other confounding factors, such as environmental and nutritional conditions, which may help to find other meaningful results. In addition, more sample data are needed, as the sample size of the subgroup is insufficient. Further validation in other cohorts is also needed to identify potential biomarkers. Importantly, basic experiments are needed to further clarify the mechanism of the potential TIR-related biomarkers screened in this study.

## Conclusions

In summary, metabolite profiling of serum and urine based on the UPLC‒MS method combined with CGM metrics was performed to provide complementary insight into the differences in T1D patients with different TIR. T1D patients with unstable GV have altered metabolites and metabolic pathways, mainly including tryptophan, vitamin B6 and purine metabolism. Further investigation of potential biomarkers may provide new insights into the mechanism of diabetes complications related to GV.

### Supplementary Information

**Additional file 1: Method S1. Table S1.** Characteristics of T1D patients and healthy control. **Table S2.** 15 characteristic metabolites identified in serum. **Table S3.** 7 characteristic metabolites identified in urine. **Table S4.** 15 characteristic metabolites identified in serum. **Table S5.** 19 characteristic metabolites identified in urine. **Table S6.** Metabolic pathway analysis. **Table S7.** The Spearman analysis of the screened metabolites related to TIR.

## Data Availability

The datasets used and/or analyzed during the current study are available from the corresponding author upon reasonable request.
