# Supplementary material for: Metabolomics analysis of serum and urine in type 1 diabetes patients with different time in range derived from continuous glucose monitoring
Source: Diabetol Metab Syndr. 2024 Jan 19;16:21. doi: 10.1186/s13098-024-01257-4 (PMC10797982; doi:10.1186/s13098-024-01257-4)
Supplement: Supplementary file 1 — Additional file 1: Method S1. Table S1. Characteristics of T1D patients and healthy control. Table S2. 15 characteristic metabolites identified in serum. Table S3. 7 characteristic metabolites identified in urine. Table S4. 15 characteristic metabolites identified in serum. Table S5. 19 characteristic metabolites identified in urine. Table S6. Metabolic pathway analysis. Table S7. The Spearman analysis of the screened metabolites related to TIR. [file 13098_2024_1257_MOESM1_ESM.docx]

**Additional file Material**

**1. Method**

**1.1. Exclusion criteria of T1D patients**

1. T1D honeymoon period.
2. Having diabetic ketoacidosis and hyperglycemic hyperosmolar syndrome within the previous 3 months.
3. Women lactating, pregnant or planning pregnant during the study period.
4. Impaired liver function: ALT or AST more than 3 times and TBIL more than 2 times the upper limit of normal.
5. Severe renal insufficiency with eGFR <30 ml/min/1.73m^2^.
6. Acute myocardial infarction within the previous 3 months.
7. Being treated with glucocorticoids (oral or intravenous) for any medical condition.
8. Anemia: haemoglobin ≤ 120 g/L in men and ≤ 110 g/L in women.
9. History of blood transfusion within the last 3 months.
10. Patients with malignancies, psychiatric disorders or serious systemic diseases that not suitable for enrollment.
11. Allergy to medical grade viscose.
12. Those requiring MRI during the study period.

**1.2. Ultra Performance Liquid Chromatography-Mass Spectroscopy (UPLC-MS) Conditions**

All stored samples were thawed at 4℃. Supernatant of 100μl and 200μl were collected from serum and urine samples with 400ul and 200μl acetonitrile (Thermo Fisher Scientific, USA) added respectively then. After vortex and placed at 4℃ for 30 minutes, samples were centrifuged at 14000g for 10 minutes. The supernatant was dissolved again with 100μl of 2% acetonitrile, centrifuged for 10 minutes at 14000g, and then filtered through 10kD membrane before UPLC-MS analysis. A Waters H-class UPLC system was used to analyze the serum and urine samples. Chromatographic separation archived on an HSS C18 column (3.0×100mm, 1.7 μm; Waters) with a column temperature at 50℃ and injection volume of 10μl. Mobile phase A and B were 0.1% formic acid in water (Thermo Fisher Scientific, USA) and acetonitrile, respectively. The flow rate was 0.3ml/min. The LC gradient was set as this: 0~2min, 2%B; 2~15min, 2%B~98%B; 15~15.1min, 98%B~100%B; 15.1~25min, 100%B; 25~25.1min, 100 %B~2%B; 25.1~30min, 2%B. UPLC was coupled with LTQ-Orbitrap velos (Thermo Fisher Scientific, SanJose, CA, USA) mass spectrometry. The MS was operated in positive ion mode using electrospray ion source. The sheath gas used nitrogen and auxiliary gas with flow rates of 45 and 10 arbitrary units respectively. The mass scanning range is 100~1000 m/z. Spray voltages of 4.2kV and capillary temperature of 350℃ were used. The MS data were collected using high-resolution Fourier Transformation with MS^1^ resolution of 60000 and MS^2^ resolution of 15000. MS^2^ adopts data-dependent analysis mode. The dynamic exclusion duration was 15s. High energy collision dissociation (HCD) was used for fragmentation with isolation width of 3Da, collision energy of 20%, 40%, and 60% according to different metabolites and activation time of 30ms. Data quality control (QC) was evaluated by the correlation of QC samples.

**2. Tables**

**Table S1. Characteristics of T1D patients and healthy control**

| **Variable** | **T1D, N=85** | **Control, N=81** | **P-value** |
| --- | --- | --- | --- |
| Gender (F/M) | 55/30 | 48/33 | 0.470 |
| Age (y.o) | 35.00(18.00) | 37.00(19.00) | 0.272 |
| Height (cm) | 1.65(0.10) | 1.66(0.15) | 0.535 |
| Weight (kg) | 57.60(12.50) | 61.40(22.30) | 0.173 |
| BMI (kg/m^2^) | 21.16(3.65) | 22.23(5.43) | 0.286 |
| HbA1c (%) | 7.40(1.75) | 5.20(0.45) | **＜0.001** |
| TC (mmol/L) | 4.63(1.14) | 4.41(1.49) | 0.878 |
| TG (mmol/L) | 0.54(0.30) | 0.95(0.74) | **＜0.001** |
| HDL-C (mmol/L) | 1.57(0.69) | 1.42(0.53) | **0.002** |
| LDL-C (mmol/L) | 2.43(0.99) | 2.75(1.07) | **0.010** |

**Table S2. 15 characteristic metabolites identified in serum (T1D vs. CON, n=81 vs.55)**

| **Query** | **HMDB** | **KEGG** | **P value** | **FC** | **log2(FC)** |
| --- | --- | --- | --- | --- | --- |
| Cortisol | HMDB0000063 | C00735 | 1.98E-08 | 1.7327 | 0.79298 |
| Neosaxitoxin | HMDB0029369 | C17208 | 3.14E-07 | 2.3562 | 1.2364 |
| Cortol | HMDB0003180 | C05482 | 5.06E-07 | 1.9005 | 0.92635 |
| METHACHOLINE | HMDB0015654 | C07471 | 9.18E-07 | 1.5979 | 0.67621 |
| 1-Methyladenosine | HMDB0003331 | C02494 | 3.14E-06 | 1.8162 | 0.8609 |
| 5-Phenyl-1,3-oxazinane-2,4-dione | HMDB0060400 | C16596 | 9.93E-06 | 1.4006 | 0.48606 |
| 5-Methoxyindoleacetate | HMDB0004096 | C05660 | 3.94E-05 | 1.6552 | 0.727 |
| Isoquinoline | HMDB0034244 | C06323 | 6.30E-05 | 1.4458 | 0.53183 |
| Deoxycholic acid glycine conjugate | HMDB0000631 | C05464 | 7.98E-05 | 2.6406 | 1.4009 |
| Chitobiose | HMDB0003556 | C01674 | 0.000235 | 1.7622 | 0.81735 |
| Glycochenodeoxycholic acid 3-glucuronide | HMDB0002579 | C03033 | 0.000899 | 2.2025 | 1.1391 |
| Sphinganine | HMDB0000269 | C00836 | 0.001094 | 0.65654 | -0.60704 |
| 4-(2-Aminophenyl)-2,4-dioxobutanoate | HMDB0000978 | C01252 | 0.001193 | 1.4062 | 0.49181 |
| 4-Pyridoxic acid | HMDB0000017 | C00847 | 0.005515 | 2.5942 | 1.3753 |
| 5-Hydroxy-L-tryptophan | HMDB0000472 | C00643 | 0.025686 | 1.3337 | 0.41539 |

| **Table S3. 7 characteristic metabolites identified in urine (T1D vs. CON, n=78 vs. 57)** | | | | | |
| --- | --- | --- | --- | --- | --- |
| **Query** | **HMDB** | **KEGG** | **P value** | **FC** | **log2(FC)** |
| 6-Hydroxy-5-methoxyindole glucuronide | HMDB0010362 | C03033 | 2.31E-05 | 0.64283 | -0.6375 |
| Petasitenine | HMDB0030328 | C10359 | 0.000375 | 15.656 | 3.9687 |
| Lucuminoside | HMDB0029900 | C08335 | 0.000532 | 12.764 | 3.674 |
| Hypoxanthine | HMDB0000157 | C00262 | 0.008679 | 0.78236 | -0.35409 |
| 4-oxo-Retinoic acid | HMDB0006285 | C16678 | 0.018749 | 1.7105 | 0.77438 |
| Withanolide B | HMDB0030020 | C00828 | 0.040601 | 1.6236 | 0.69921 |
| N6,N6,N6-Trimethyl-L-lysine | HMDB0001325 | C03793 | 0.047546 | 1.7088 | 0.77301 |

**Table S4. 15 characteristic metabolites identified in serum (TIR-L vs. TIR-H, n=20 vs.12)**

| **Query** | **HMDB** | **KEGG** | **VIP value** | **P value** | **FC** | **log2(FC)** | |
| --- | --- | --- | --- | --- | --- | --- | --- |
| Mevalonolactone | HMDB0006024 | NA | 2.6453 | 0.0002 | 0.2811 | -1.8308 |  |
| N-Phenylacetylaspartic acid | HMDB0029355 | NA | 2.2188 | 0.0425 | 0.4138 | -1.2732 |  |
| Hexadecanedioic acid mono-L-carnitine ester | HMDB0000712 | NA | 1.8236 | 0.0649 | 1.8168 | 0.8614 |  |
| 4-Pyridoxic acid | HMDB0000017 | C00847 | 1.8041 | 0.1088 | 0.5035 | -0.9901 |  |
| 5-Hydroxy-L-tryptophan | HMDB0000472 | C00643 | 1.6627 | 0.0400 | 0.6398 | -0.6442 |  |
| 11-Methoxynoryangonin | HMDB0030754 | NA | 1.4115 | 0.0486 | 1.8732 | 0.9055 |  |
| Isoquinoline | HMDB0034244 | C06323 | 1.3442 | 0.0569 | 0.7347 | -0.4449 |  |
| L-beta-aspartyl-L-serine | HMDB0011168 | NA | 1.3370 | 0.1134 | 0.7166 | -0.4808 |  |
| Tyrosyl-Glycine | HMDB0029105 | NA | 1.2788 | 0.3868 | 0.8491 | -0.2360 |  |
| 1-(1,2,3,4,5-Pentahydroxypent-1-yl)-1,2,3,4-tetrahydro-beta-carboline-3-carboxylate | HMDB0012492 | NA | 1.2537 | 0.0506 | 1.7039 | 0.7688 |  |
| Chitobiose | HMDB0003556 | C01674 | 1.2366 | 0.7360 | 0.9126 | -0.1320 |  |
| Deoxycholic acid glycine conjugate | HMDB0000631 | C05464 | 1.2084 | 0.1129 | 2.2031 | 1.1396 |  |
| Glutamylisoleucine | HMDB0028822 | NA | 1.1749 | 0.1596 | 2.7418 | 1.4551 |  |
| Isoleucyl-Hydroxyproline | HMDB0028908 | NA | 1.0603 | 0.1676 | 1.8989 | 0.9252 |  |
| Neosaxitoxin | HMDB0029369 | C17208 | 1.0024 | 0.2591 | 1.3264 | 0.4075 |  |

**Table S5. 19 characteristic metabolites identified in urine (TIR-L vs. TIR-H, n=19 vs.14)**

| **Query** | **HMDB** | **KEGG** | **VIP value** | **P value** | **FC** | **log2(FC)** |
| --- | --- | --- | --- | --- | --- | --- |
| (1(10)E,4E,6a,9b)-9-(2-Methylpropanoyloxy)-1(10),4,11(13)-germacratrien-12,6-olide | HMDB0031373 | NA | 1.7586 | 0.0161 | 1.6186 | 0.6948 |
| Thromboxane B3 | HMDB0005099 | NA | 1.5800 | 0.0245 | 0.6146 | -0.7022 |
| (E)-1-O-Cinnamoyl-beta-D-glucose | HMDB0030293 | NA | 1.4383 | 0.0080 | 4.1447 | 2.0513 |
| Cynaratriol | HMDB0034983 | NA | 1.3914 | 0.0714 | 1.9787 | 0.9846 |
| Prehumulinic acid | HMDB0030148 | NA | 1.3797 | 0.1221 | 1.4116 | 0.4973 |
| Gibberellin A54 | HMDB0035048 | NA | 1.3440 | 0.0274 | 5.5583 | 2.4746 |
| 1-(1,2,3,4,5-Pentahydroxypent-1-yl)-1,2,3,4-tetrahydro-beta-carboline-3-carboxylate | HMDB0012492 | NA | 1.3314 | 0.0134 | 4.9495 | 2.3073 |
| Phenylbutyrylglutamine | HMDB0011687 | NA | 1.3021 | 0.0267 | 0.2090 | -2.2585 |
| 15-Octadecene-9,11,13-triynoic acid | HMDB0032673 | NA | 1.2941 | 0.0114 | 7.2922 | 2.8664 |
| Lucuminoside | HMDB0029900 | C08335 | 1.2404 | 0.0257 | 6.6548 | 2.7344 |
| Humulinic acid A | HMDB0030104 | NA | 1.2370 | 0.1466 | 1.3308 | 0.4123 |
| 3b-Allotetrahydrocortisol | HMDB0000314 | NA | 1.2322 | 0.0187 | 1.8611 | 0.8962 |
| Petasitenine | HMDB0030328 | C10359 | 1.2041 | 0.0181 | 7.0353 | 2.8146 |
| Cinnzeylanol | HMDB0036010 | NA | 1.1806 | 0.0519 | 5.5465 | 2.4716 |
| N6,N6,N6-Trimethyl-L-lysine | HMDB0001325 | C03793 | 1.1390 | 0.0547 | 0.4269 | -1.2279 |
| Sinapoylputrescine | HMDB0033464 | NA | 1.0897 | 0.0494 | 0.5824 | -0.7799 |
| Hypoxanthine | HMDB0000157 | C00262 | 1.0109 | 0.0638 | 0.7261 | -0.4618 |
| 4-oxo-Retinoic acid | HMDB0006285 | C16678 | 1.0027 | 0.0614 | 1.7273 | 0.7885 |
| Hydroxyprolyl-Lysine | HMDB0028868 | NA | 1.0009 | 0.0397 | 7.1409 | 2.8361 |

**Table S6. Metabolic pathway analysis**

| **Pathway** | **Total** | **Hits** | **Compounds** | **P value** | **-LOG10(p)** | **Impact** |
| --- | --- | --- | --- | --- | --- | --- |
| T1D vs. CON (serum) | | | | | | |
| Tryptophan metabolism | 41 | 3 | 5-Hydroxy-L-tryptophan,  5-Methoxyindoleacetate,  4-(2-Aminophenyl)-2,4-dioxobutanoate | 4.15E-05 | 4.3824 | 0.13094 |
| Vitamin B6 metabolism | 9 | 1 | 4-Pyridoxate | 0.005515 | 2.2585 | 0 |
| Pentose and glucuronate interconversions | 18 | 1 | Glycochenodeoxycholic acid 3-glucuronide | 0.000899 | 3.0461 | 0.14062 |
| Sphingolipid metabolism | 21 | 1 | Sphinganine | 0.001094 | 2.9609 | 0.15416 |
| Amino sugar and nucleotide sugar metabolism | 37 | 1 | Chitobiose | 0.000235 | 3.6293 | 0 |
| Drug metabolism-cytochrome P450 | 55 | 1 | 5-Phenyl-1,3-oxazinane-2,4-dione | 9.93E-06 | 5.0031 | 0 |
| Steroid hormone biosynthesis | 85 | 1 | Cortisol | 1.98E-08 | 7.7034 | 0.02729 |
| T1D vs. CON (urine) | | | | | | |
| Pentose and glucuronate interconversions | 18 | 1 | 6-Hydroxy-5-methoxyindole glucuronide | 2.31E-05 | 4.6371 | 0.14062 |
| Purine metabolism | 65 | 1 | Hypoxanthine | 0.008679 | 2.0616 | 0.01651 |
| Ubiquinone and other terpenoid-quinone biosynthesis | 9 | 1 | Withanolide B | 0.040601 | 1.3915 | 0 |
| Lysine degradation | 25 | 1 | N6,N6,N6-Trimethyl-L-lysine | 0.047546 | 1.3229 | 0 |
| TIR-L vs. TIR-H (serum) | | | | | | |
| Tryptophan metabolism | 41 | 1 | 5-Hydroxy-L-tryptophan | 0.039967 | 1.3983 | 0.13094 |
| Vitamin B6 metabolism | 9 | 1 | 4-Pyridoxate | 0.1088 | 0.96336 | 0 |
| Amino sugar and nucleotide sugar metabolism | 37 | 1 | Chitobiose | 0.73597 | 0.13314 | 0 |
| TIR-L vs. TIR-H (urine) | | | | | | |
| Lysine degradation | 25 | 1 | N6,N6,N6-Trimethyl-L-lysine | 0.054676 | 1.2622 | 0 |
| Purine metabolism | 65 | 1 | Hypoxanthine | 0.063833 | 1.195 | 0.01651 |

**Table S7. The Spearman analysis of the** **screened metabolites related to TIR**

| **Metabolite** | **HMDB** | **log2(FC)** | **P value** | **VIP** | **R^#^** | **P^#^** | **R*** |
| --- | --- | --- | --- | --- | --- | --- | --- |
| **Serum** | | | | | | | |
| Mevalonolactone | HMDB0006024 | -1.8308 | <0.001 | 2.6453 | **0.367** | **0.001** | **0.320** |
| L-beta-aspartyl-L-serine | HMDB0011168 | -0.4808 | 0.113 | 1.3370 | **0.222** | **0.046** | - |
| **Urine** | | | | | | | |
| Hypoxanthine | HMDB0000157 | -0.4618 | 0.064 | 1.0109 | **0.244** | **0.032** | **0.232** |
| Phenylbutyrylglutamine | HMDB0011687 | -2.2585 | 0.027 | 1.3021 | **0.329** | **0.003** | **0.308** |
| Gibberellin A54 | HMDB0035048 | 2.4746 | 0.027 | 1.3440 | **-0.521** | **<0.001** | - |
| (E)-1-O-Cinnamoyl-beta-D-glucose | HMDB0030293 | 2.0513 | 0.008 | 1.4383 | **-0.462** | **<0.001** | - |
| 1-(1,2,3,4,5-Pentahydroxypent-1-yl)-1,2,3,4-tetrahydro-beta-carboline-3-carboxylate | HMDB0012492 | 2.3073 | 0.013 | 1.3314 | **-0.438** | **<0.001** | - |
| Lucuminoside | HMDB0029900 | 2.7344 | 0.026 | 1.2404 | **-0.399** | **<0.001** | - |
| Cinnzeylanol | HMDB0036010 | 2.4716 | 0.052 | 1.1806 | **-0.385** | **<0.001** | - |
| 15-Octadecene-9,11,13-triynoic acid | HMDB0032673 | 2.8664 | 0.011 | 1.2941 | **-0.383** | **0.001** | - |
| Petasitenine | HMDB0030328 | 2.8146 | 0.018 | 1.2041 | **-0.262** | **0.020** | - |
| (1(10)E,4E,6a,9b)-9-(2-Methylpropanoyloxy)-1(10),4,11(13)-germacratrien-12,6-olide | HMDB0031373 | 0.6948 | 0.016 | 1.7586 | **-0.237** | **0.036** | - |
| Log2(FC), P value and VIP are calculated by comparing TIR-L with TIR-H group.  # R and P represents the correlation value and p value by spearman analysis. R* represents the correlation value by partial correlation analysis when adjusting for gender, age, BMI, duration of disease and insulin dosage. Bold text represents the correlation is significant (P<0.05). | | | | | | | |
